# IgG-k/IgG-λ Para-Osseous Plasmacytoma Relapsed as Soft-Tissue Plasmacytoma with IgA-k Immunophenotype: A Case Report and Review of the Literature on Related Biochemical Aspects

**DOI:** 10.3390/hematolrep16030052

**Published:** 2024-08-29

**Authors:** Manlio Fazio, Chiara Maria Catena Sorbello, Vittorio Del Fabro, Alessandra Romano, Maria Teresa Cannizzaro, Nunziatina Laura Parrinello, Benedetta Esposito, Sara Frazzetto, Federica Elia, Francesco Di Raimondo, Concetta Conticello

**Affiliations:** 1 Post-Graduation School of Haematology, University of Catania, A.O.U. ‘Policlinico-San Marco’, 95123 Catania, Italy; chiara_sorbello@hotmail.it (C.M.C.S.); benedetta.esposito89@gmail.com (B.E.); diraimon@unict.it (F.D.R.); 2 Division of Haematology and BMT, A.O.U. ‘Policlinico-San Marco’, 95123 Catania, Italy; vdelfabro@yahoo.it (V.D.F.); alessandra.romano@unict.it (A.R.); parrinellolaura@gmail.com (N.L.P.); fede.elia31@gmail.com (F.E.); 3 Dipartimento di Specialità Medico-Chirurgiche, Dipartimento di Chirurgia Generale e Specialità Medico-Chirurgiche, Sezione di Ematologia, Università degli Studi di Catania, 95131 Catania, Italy; 4 Radiology via Santa Sofia 78 AOU Policlinico—“Vittorio Emanuele”, 95123 Catania, Italy; maratex@yahoo.it

**Keywords:** extramedullary myeloma, para-osseus and soft-tissue plasmacytoma, relapsed-refractory, bi-phenotype and bi-clonal myeloma, free light chains, IgA monoclonal component, target antigens, therapeutic sequencing, risk sub-category

## Abstract

Neoplastic plasma cells (PCs) proliferation at anatomic sites dislocated from the bone marrow (BM) or their contiguous growth from osseous lesions that disrupt the cortical bone is termed extramedullary multiple myeloma (EMD). EMD still remains challenging from a therapeutic and biological perspective. Pathogenesis has not been completely clarified, and it is generally associated with high-risk cytogenetics (HRCAs). In order to emphasize the clinical and biochemical complexity of this disease, we have decided to describe the case of a patient affected by relapsed-refractory (RR) EMD, which presented as para-osseous plasmacytoma with a bi-phenotypical immunoglobulin (Ig) component and lately relapsed as soft-tissue plasmacytoma with a total immunophenotype switch. We have also hypothesized a correlation between Ig patterns and prognosis and suggested the possible inclusion of these biochemical features in the general risk assessment.

## 1. Introduction

Extra-medullary multiple myeloma (EMD) is a tumor mass derived from an abnormal proliferation of neoplastic plasma cells (PCs) outside the bone marrow microenvironment. Various clinical entities belong to this very aggressive pathological category and can be distinguished according to the tissue involved: para-osseous plasmacytoma (PO); solitary plasmacytoma (SP); and extramedullary plasmacytoma (EMP). More specifically, PO is a mass of myelomatous PCs that thrives exophitically from the bone (disrupting the cortical layer); SP is a single tumoral growth of PCs, involving either the bone or EM sites, which is not associated with the contemporary presence of neoplastic PCs in the BM and whose symptoms are caused by expansion; on the contrary, the last entity, which is EMP, is a tumor mass prospering in organs/soft tissues as the result of the hematogenous spread of clonal PCs originally localized in the BM [1]. Possible mechanisms that can justify PCs escaping from the BM microenvironment include the expression of the C-X-C motif chemokine receptor 4 (CXCR4) that acts through its ligand CXCL12. In addition to its role in plasma cells (PCs) homing, the CXCR4/CXCL12 axis is also involved in PCs mobilization, egression, and dissemination out of the BM [2].

EMD can occur at any site. At diagnosis, it is typically found in skin and soft tissues; at relapse, typical sites involved include the liver, kidneys, lymph nodes, central nervous system (CNS), breast, pleura, and pericardium. Improvements in diagnostic assessments have led to a more frequent detection of the disease. At present, in patients with newly diagnosed MM, the reported incidence ranges from 0.5% to 4.8% (primary EMD), while in relapsed/refractory MM, the reported incidence is 3.4 to 14% (secondary EMD) [1]. In both cases, it identifies an aggressive disease, often characterized by unfavorable prognostic features such as high-risk cytogenetic aberrations (HRCAs) [3], mutations of individual genes, epigenetic changes [4], increased proliferation, and drug resistance.

Other factors that portend poor outcomes in the case of EMD are dissemination from BM rather than direct growth from the bone, involvement of >1 organ, no ASCT, no CR after ASCT, high β2-microglobulin levels (>5 mmol/L), ISS II and III status, and acute graft versus host disease (GVHD) after alloSCT. Furthermore, a retrospective study by Shin et al. suggested that a low platelet (PLT) count at diagnosis and BM PCs% after SCT have a negative impact on prognosis (but further studies are needed to confirm this statement) [5].

Lastly, central nervous system (CNS) involvement represents a particularly rare (0.7%) and aggressive EMD subcategory. More specifically, OS with leptomeningeal involvement is nearly 6 months [6]. In this regard, immunomodulatory drugs (IMiDs) such as pomalidomide or thalidomide and proteasome inhibitors (PI) such as marizomib have shown major activity on the CNS, due to their capability to cross the blood–brain barrier (BBB) [7]. Radiotherapy and intrathecal chemotherapy are other possible alternatives.

Positron emission tomography/computer tomography (PET/CT) is an essential imaging technique both at diagnosis and for response assessment throughout treatment. It has proven to be effective both in identifying EMD and in predicting the evolution from asymptomatic to symptomatic disease [8]. According to the new IMWG diagnostic criteria, the presence of metabolically active lesions is itself a sufficient *criterium* to start a chemo-immunotherapy regimen. PET/CT also correlates with prognosis since extensive and elevated standardized uptake volume (SUV) are generally associated with more aggressive disease [9]. A tissue biopsy should also be performed at diagnosis in order to evaluate the presence and percentage of PCs [10]. Generally, patients who develop EMD lately in the course of the disease have lower levels of serum monoclonal components, lower Hb, and higher lactate dehydrogenase (LDH) levels compared to EMD at diagnosis. 

Another aspect that should be evaluated in terms of prognosis is the immunophenotype of the monoclonal component (MC). There are some reports in the literature describing MM and EMD patients who present with a double-MC or undergo an immunophenotype switch at relapse [11,12,13,14,15]. This suggests the development of a new aberrant clone, potentially more aggressive than the original one. Reports in the literature have tried to investigate the prognostic value of these peculiar biochemical characteristics. However, results are often discordant and limited to a small cohort of patients. In order to address this issue, we have decided to describe the therapeutic journey of a relapsed-refractory patient affected by EMD whose disease was characterized by both biochemical features (a bi-phenotypic isotype at diagnosis and an isotype switch at relapse). This allowed us to provide a detailed review of the literature regarding peculiar immunoglobulin patterns in multiple myeloma, particularly extramedullary disease, and suggest a possible correlation with prognosis. Lastly, we hope to stimulate further studies that will shed light on the molecular processes that lie underneath the pathogenesis of the disease and will suggest new pathways to treat this obstinate, incurable neoplasm.

## 2. Case Description

We describe the case of a 59-year-old Caucasian man affected by multiple myeloma (MM) with a double monoclonal immunoglobulin (IgG) component with k and λ chains. 

In 2019, he was initially diagnosed with para-osseous (PO) plasmacytoma involving the eight thoracic vertebra (Th8) and was assessed as stage IIIA according to Durie and Salmon and stage I according to the International Staging System (ISS). Laboratory exams showed serum k-free light chains (FLCs) 49.8 mg/L [normal range (n.r.) 6.7–22.4 mg/L], λ-FLC 41.3 mg/L (n.r. 8.3–27 mg/L), serum k/λ ratio 1.21, serum electrophoresis demonstrated a double monoclonal component (MC) (2.8–0.22% g/dL and 3.3–0.26% gr/dL); urine k-FLC were 19.20 mg/dL and λ-FLC 2.22 mg/dL and urine immunofixation (IFE) showed a k and λ MC both Bence Jones (Bj) positive; creatinine, serum calcium levels and blood count were in normal range. 

Fluorescence in situ hybridization (FISH) analyses revealed the presence of high-risk cytogenetic abnormalities (HRCAs): deletion of chromosome 13q (13q⁻) and gain of chromosome 1q (+1q). He had subsequently undergone a neurosurgical operation in order to obtain vertebral decompression and started 1st line treatment with bortezomib, thalidomide, and dexamethasone (VTd), administered for 4–28-day cycles. 

After apheretical stem cell collection, he received a **double autologous stem-cell transplant (ASCT)**, followed by monthly lenalidomide maintenance (LenMT) at 10–15 mg/day (days 1–21). After the 2nd ASCT, the patient reached a complete response (CR) according to the international Myeloma Working Group (IMWG) criteria (negative immunofixation on both serum and urine and <5% PCs in the BM), despite interrupting treatment several times due to cutaneous toxicity.

**Figure 1:**
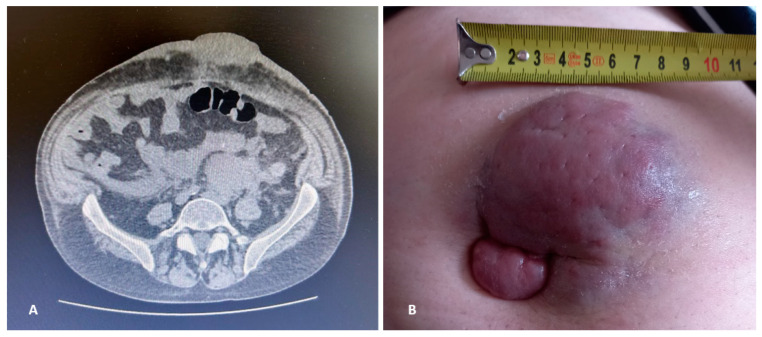
**Radiological and clinical images.** CT scan of the abdomen: tissue elevation of about 43 × 28 mm at the peri-umbilical site involving the soft tissues of the left anterior abdominal wall and extended to the para-umbilical region, characterized by pseudo-nodular morphology (**A**). Umbilical and para-umbilical lesion protruding from the umbilical scar: 80 × 60 mm, raised about 2 cm, purplish colored and with hard-rubbery consistency (**B**).

In 2022, he faced his first relapse. He presented with new bone lesions. Serum protein electrophoresis revealed an IgG isotype switch to an IgA-k monoclonal component (30.7–2.61% g/dL) detected in the β1 region; serum k-FLC were 256 mg/L; urine IFE was also positive for Bj-positive k-MC. Having not employed an anti-CD38 monoclonal antibody (mAb) in induction, we decide to treat the patient with **daratumumab–bortezomib–dexamethasone (DV-d scheme)**, achieving a PR according to IMWG (>50% reduction in serum MC, which turned 1.12 gr/L). In February 2023 (after the 5th cycle), he experienced the 2nd relapse (IgA-k MC 43–3.57% g/dL; serum k-FLC 226 mg/dL; k/λ 24.41; while creatinine and calcium were still in range). At physical exam of the umbilical and para-umbilical areas, we documented the presence of a dome-shaped lesion with the following characteristics: protrusion from the umbilical scar, height 2 cm, width 8 cm, purplish color, and hard-rubbery consistency (Figure 1B). Therefore, we performed a computer tomography (CT) scan of the abdomen, which documented the presence of expanding tissue measuring 55 mm × 45 mm that could not be dissociated from the adjoining intestinal loops of the duodenum–jejunal tract. Another tissue formation measuring 43 × 28 mm and displaying a pseudo-nodular aspect contextually involved the peri-umbilical area, soft tissues of the left anterior abdominal wall, and the para-umbilical region (Figure 1A).

**Figure 2:**
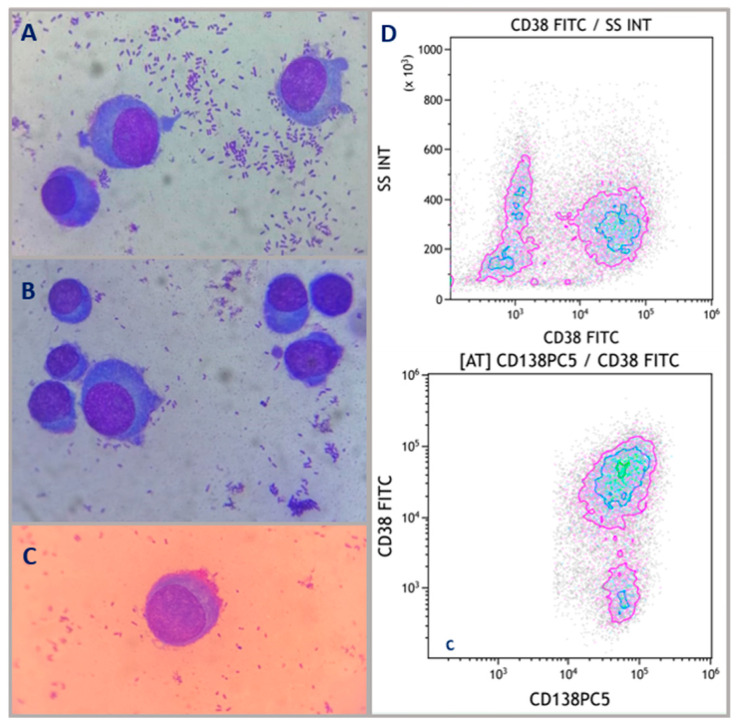
**Morphological images and flow cytometry.** Peripheral blood smear aspirated from the para-umbilical lesion: hypercellularity primarily represented by PCs (70%). PCs cytoplasm is quite basophilic; there is a lighter area inside that corresponds to Golgi apparatus and centrioles. The nucleus is usually localized eccentrically with heterochromatin arranged in a characteristic wagon wheel feature (**A**–**C**). Cytometry analyses of the material obtained from the lesion show the almost total presence of PCs with a CD138+ and partly CD38+ immunophenotype (**D**).

We decided to perform a blood aspiration from the lesion. At optic microscopy analysis, we found a cellular population ubiquitously represented by plasma cells (PCs) (Figure 2A–C). Flowcytometry analyses confirmed a CD138+ and CD38+/− immunophenotype (Figure 2D). 

**Figure 3:**
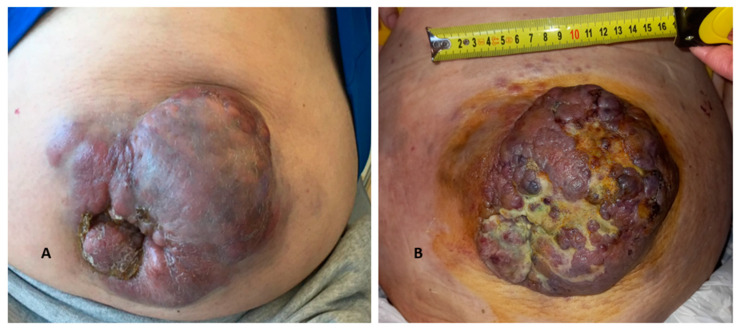
**Increase in the size of soft-tissue plasmacytoma and evolution of the lesion.** After 6 cycles of Elo-Pd as 3rd line of therapy, the patient had to interrupt treatment due to new progression and extension of the abdominal lesion. Cross diameters measured about 10 × 15 cm, and the whole formation raised about 4 cm from the abdominal plane. As shown in the picture, it appeared lumpy, irregular, and purplish. At physical exam, it presented hard-rubbery consistency (**A**). Central fibrotic degeneration after DCEP polychemotherapy (**B**).

Therefore, we decided to change target and employ the anti-SLAMF7 (signaling lymphocytic activation molecule family member 7) monoclonal antibody **elotuzumab associated with pomalidomide (EloPd)**. We managed to obtain a PR again, with a nearly 75% MC reduction (10.2–0.72% g/dL) and >50% decrease in the k/λ ratio, and the tissue lesion remained stable. After only 6 cycles, we documented disease progression (45.9–4.27% IgA MC; serum k-FLC 270 mg/L). The lesion became wider (10 cm wide and 15 cm long), higher (4 cm from the abdominal surface), lumpy, and discolored (Figure 3A)**.**

In July 2023, we started the IV line of therapy employing the regimen **isatuximab–carfilzomib–dexamethasone (Isa-Kd)**. Treatment was soon interrupted due to immediate disease progression (MC 49.8–4.58% g/dL; serum k-FLC 530 mg/L; λ-FLC 8.67 mg/L; k/λ 61.13; creatinine 1.73 mg/dL; while calcium was nearly in range) associated with pancytopenia (Hb 7.15 g/dL, PLT 29.630/m^3^, WBC 4540/m^3^) and abnormal lactate dehydrogenase levels (567 U/L-n.r. 0–248 U/L). Both serum and urine immunofixation confirmed the persistence of an IgA-k monoclonal component.

**Figure 4:**
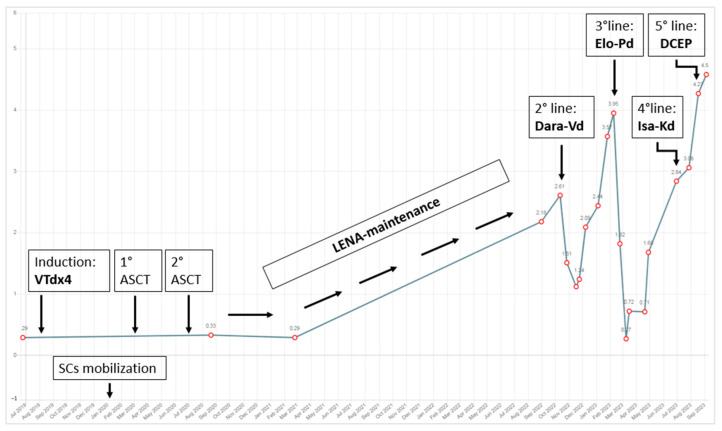
**Graph showing the trend of the monoclonal component throughout the years.** ABBREVIATIONS: VTd (bortezomib–thalidomide–dexamethasone); ASCT (autologous stem cells transplant); SCs (stem cells); LENA (lenalidomide); Dara-Vd (daratumumab–bortezomib–dexamethasone); Elo-Pd (elotuzumab–pomalidomide–dexamethasone); Isa-Kd (isatuximab–carfilzomib–dexamethasone); DCEP (dexamethasone–cyclophosphamide–etoposide–cisplatin).

The patient was ultimately hospitalized in order to receive a red blood cell (RBC) transfusion, granulocyte colony-stimulating factor (GCSF) administration, and to be initiated on lymphoma-like poly-chemotherapy with **dexamethasone–cyclophosphamide–etoposide–cisplatin (DCEP)**. The patient reached PR after 2 months (IgA-k MC was 24.3–1.34% g/dL; serum k-FLC 80.1 mg/L; k/λ ratio 4.31; IgA-k in β region at serum IFE; creatinine 1.20 mg/dL), and the cutaneous lesion underwent an initial involution by progressive sclerosis of the central area (Figure 3B). Unfortunately, he developed several opportunistic infections during hospitalization and recently passed away due to septic and hemorrhagic complications. A graph showing the trend of the monoclonal component is described in Figure 4.

## 3. Discussion

### 3.1. Simultaneous Presence or Consecutive Development of a Second Monoclonal Component: Peculiar Immunoglobulin Patterns and Their Clinical and Prognostic Significance

Normal plasma cells are terminally differentiated effector B cells developed from naïve marginal zone B cells and follicular B cells after an antigen encounter. The immunoglobulins produced by normal plasma cells are central to the body’s adaptive immune response to foreign antigens. Immunoglobulins are either secretory or cell surface-bound proteins that are composed of two heavy chains (α, γ, δ, ε, or μ) and two light chains (κ or λ). Neoplastic plasma cells in the majority of plasma cell myeloma cases retain the ability to produce either complete immunoglobulins or at least a fragment of paraproteins [16]. In fact, according to a mechanism of allelic exclusion, immunoglobulin production is the result of sequential locus rearrangements (1° heavy chains, 2° k-FLC, and 3° λ-FLC). If clonal PCs fail the allelic exclusion process, this can lead to a lack of heavy chains and further k and λ LC gene rearrangements. Plasma cell myelomas produce or express different paraproteins. The frequency of these paraproteins is as follows: IgG (60%), IgA (24%), IgD (3%), IgM (0.5%), IgE (very rare), light chain only myeloma (11%), and non-secretory myeloma (less than 1%). Up to 2% of myeloma cases are also found to secrete more than one paraprotein, of which the majority secrete two different heavy chain isotypes or subclasses. These myeloma cases are classified as bi-clonal plasma cell myeloma [17]. There are very few reported cases in the literature in which myeloma cells are found to express both kappa and lambda light chains in a single clonal plasma cell population.

#### 3.1.1. Bi-Phenotypical/Bi-Clonal Paraproteinemia: A Possible Risk Sub-Category?

In the case described in this article, the patient presented with a double IgG-k/IgG-λ monoclonal component. Bi-phenotypical multiple myeloma is a very rare entity, and there are few real-life studies aiming to evaluate its prognostic significance on a larger scale. It may derive from the independent proliferation of two separate clones of PCs or from a single clone of PCs producing two different CMs [18]. 

A study published in 1981 analyzed 57 patients with bi-clonal gammopathy (from 1966 to 1979). Patients affected by MM were only nine (16%). Of these nine patients, six had IgG and IgA bi-clonal gammopathy, and one each had lgA-K and IgA-λ, IgG-λ and IgG-k, and IgA-λ and IgE-λ. One patient had osteosclerotic myeloma and severe peripheral neuropathy, and another had plasma-cell leukemia; one had a solitary plasmacytoma of the ilium for six years, and then disseminated myeloma developed; another had been treated for IgA-k MM for 8 years, and then an extra IgA-λ CM appeared (this was not associated with an acceleration of the disease). The study concludes by reporting that no significant difference has been found between bi-clonal and monoclonal disease in terms of response to therapy and survival. Lastly, authors justify the pathogenesis of bi-clonal gammopathies with a second independent event that leads to another cell clone formation. Other alternative hypotheses are (1) the occurrence of a transforming event due to a strong antigenic stimulus that expands antigen-reactive clones of plasma cells; (2) the occurrence of a transforming event in cells undergoing a switch from one heavy chain class to another [19].

Several case reports populate the scientific literature describing the course and outcome of bi-phenotypic gammopathies [11,12,13]. A case series published in 2018 described three patients with bi-phenotypical features. The first patient (65-year-old male) had IgA MM (4481 mg/dL) with k and λ free light chains dual expression and presented with anemia and bone lesions. The karyotype was normal. Monosomy 13 was detected by fluorescent in situ hybridization (FISH). A single clonal plasma cell population was demonstrated by immunoglobulin heavy chain (IgH) polymerase chain reaction (PCR), which detected a clonal IgH gene rearrangement with a single high peak. The second patient (58-year-old male) had a monoclonal IgG-λ MM at diagnosis and was treated with Rd-ASCT, obtaining CR. He relapsed 8 years later, presenting with bone lesions, anemia, and sFLC double expression. Karyotype was complex with several structural alterations. The third patient (76-year-old female) presented with eosinophilia and increased LDH levels. A BM biopsy was performed, and immunohistochemistry (IHC) stains and mRNA in situ hybridization (ISH) tests confirmed that PCs were both k+ and λ+. The karyotype was normal; the FISH detected 1q+, +5, and 13q-. On the basis of cytogenetic or FISH features harbored by these patients, the authors emphasized that bi-phenotypic MM could correlate with a higher risk class and an unfavorable prognosis. Despite the small number of cases, considering that our patient was originally diagnosed with parosseous plasmacytoma with FISH positivity for del 13q and +1q, we further support this hypothesis [20].

#### 3.1.2. Patient’s Relapse with IgA-k Switch: Role of sFLC Assay in R/R MM and Prognostic Significance of IgA MC

In a retrospective study, Markovic et al. analyzed the role of sFLC ratio (sFLCr) and involved sFLC at disease relapse in a real-life single-center cohort of 130 MM patients treated with ≥3 LOTs. More specifically, in order to predict populations with poorer outcomes, pre-FLC values (sFLCs assays before every LOT) were compared to the PFS status of each treatment line. On the other hand, in order to predict patients with a higher probability of clinical relapse (according to sFLC value), post-sFLC values (evaluated after every LOT) were compared with relapse type (biochemical vs. clinical). By regular monthly monitoring, the first sFLC alteration was mainly observed 6 months before confirmed disease relapse. The sFLC increase was accompanied or succeeded by an M-protein increase in more than half of the patients, while in the rest, the sFLC assay was the only serological predictor of disease relapse. On the other side, more than 20% of patients showed an oligo-secretory/micromolecular escape at relapse. Data obtained from the study demonstrated that pre- and post-sFLC >138 mg/mL and pre- and post-sFLCr >25 mg/dL have a negative impact on prognosis with a major probability of clinical relapse. Lastly, only 4/130 patients had extramedullary disease at diagnosis, while 10 had extramedullary relapse. Considering the extremely limited EMD patient sub-cohort, neither pre- nor post-sFLC values (at the abovementioned cut-offs) demonstrated a statistically significant impact in terms of PFS [21].

In our case, the patient maintained a CR for 2 years before relapsing with extramedullary plasmacytoma with IgA-k CM (serum k-FLC were 256 mg/L).

A study conducted at the Qingdao Central Hospital, First Hospital of Jilin University, and Beijing Chao-Yang Hospital (West) enrolled 129 patients (75 men and 54 women) with IgA MM to evaluate the clinical characteristics and prognosis of this type of disease. The median age was 62.9 years. Interestingly, 31.7% of patients presented with EMP (nine of these cases were associated with pleural effusion). Interphase FISH confirmed chromosome 1q21 gain, (17p) deletion, and (4;14) translocation in 45.9%, 27.8%, and 9.8% of patients, respectively. Eighty-nine (69.0%) patients received bortezomib-containing regimens [such as bortezomib plus cyclophosphamide and dexamethasone (VCd), Vd, and bortezomib plus doxorubicin and dexamethasone (PAD)]; 40 patients were treated with conventional regimens. After 25 months of follow-up, patients who received V-based regimens obtained a higher PFS (22 months vs. 10) and OS (42.0% vs. 36.0%). The ORR of this group was 95.5% (vs 55.0%), including 52.8% CR, 16.8% VGPR, and 23.6% PR. These data suggest that IgA-type MM is an unfavorable prognostic disease, often characterized by HRCAs and EMP (like in the case described in this article). However, employing bortezomib can significantly improve the outcome [22]. 

It should be specified that while the study focuses only on NDMM patients, our case report describes the occurrence of an IgA-k EMP at relapse. Extramedullary disease manifestations at relapse are particularly difficult to treat, and the prognosis is dismal. In line with the study, we treated our patient with a scheme consisting of bortezomib and dexamethasone with the addition of the anti-CD38 antibody daratumumab (DVd). As expected, our patient faced a precocious relapse after just 1 year.

This second relapse was characterized by a progressive and massive cutaneous involvement of the abdomen, as described above in the case presentation. IgA-k MC was still present. 

In a review published by Kato in 1999, a total of 83 cases of MM associated with cutaneous plasmacytomas were included. Only five of them were associated with IgA-k. However, they were specifically identified as *metastatic* cutaneous plasmacytomas, and the prognosis was generally poor [23]. Metastatic cutaneous plasmacytoma is characterized by a wide variety of skin manifestations: eruption of hard, pinkish-colored, bean-sized nodules; hard, violet-colored, dome-shaped, nodules; ecchymoses (without associated thrombocytopenia); pyoderma gangrenosum; leukocytoclastic vasculitis; vesciculo-bollous disorders [24]. However, none of these clinical entities resembles our patient’s lesions. Considering that our patient had one voluminous and protruding localized lesion, we would describe this tissue involvement as local cutaneous infiltration originating from the abdomen rather than metastatic dissemination.

#### 3.1.3. Immunoglobulin Isotype Switch: Association with EMD and Correlation with IMiDs

In a study held by the Chinese group at the Beijing Jishuitan Hospital, a total of 506 NDMM patients were followed from February 2005 to February 2020. The intent was to evaluate the clinical and prognostic significance of the immunoglobulin isotype switch at relapse. Relapsed patients were divided into four groups according to Ig phenotype: original paraprotein, complete isotype switching, light chain escape (LCE), and non-secretory clinical relapse. Focusing on the second group, 13 patients exhibited a complete isotype switch (as happened in our patient). Of these 13 patients, 1 switched from IgM-k to λ-FLC only; 7, respectively, switched from k FLC only (2 patients)/IgG-k/IgD-k/IgA-λ/IgA-k (2 patients) to non-secretory MM; 1 switched from IgD-λ to IgG-k with P53 deletion; 2 switched to k-FLC only from original IgA-k and IgG-k paraprotein; 1 switched from non-secretory MM to λ-FLC only. Interestingly, despite the impressive number of patients evaluated, none of them switched from IgG-k/λ or from a bi-phonotype to the IgA isotype. This proves the rarity of the case described in this paper. Interestingly, the majority of these patients (8/13) relapsed with an extra-medullary disease (multiple tumors/intracranial/right upper arm/lumbar/mandibular/maxillary/pelvic/shin) and were treated with lymphoma-like poly-chemotherapy regimens such as DCEP (the one we employed at last in our patient). Only 3 patients achieved CR, 1 VGPR, 3 PRs, and 1 progressive disease (PD). The one who faced PD originally was an IgA isotype (λ) MM. Of the total 13 patients, 7 survived for at least 20 months after relapse. The median OS of those who obtained CR (5 patients) was 76 months. These data proved that the prognosis in patients with isotype change does not significantly differ from that in patients without change. However, considering the difference in clinical manifestations between initial diagnosis and relapse in these 13 patients, the study provided direct clinical evidence of the heterogeneity of MM clonal populations occurring in the evolution of the disease [14].

The occurrence of an immunoglobulin isotype switch in extramedullary myeloma has not been frequently reported in the literature. An interesting case series published in 2007 described 3 patients with intact immunoglobulin (II) MM (2 with IgA-k and 1 with IgG-λ) who relapsed in extramedullary sites with a free light chain escape (LE) [15]. 

Considering these biochemical features at relapse, none of these cases perfectly match our patient. However, to some extent, we can find a certain therapeutic correspondence. More specifically, these three patients received thalidomide or lenalidomide as induction therapy. The same IMiDs were also employed in our case (VTd induction + double ASCT + LENA maintenance). Various reports in the literature have documented the appearance of extramedullary plasmacytomas at relapse after the employment of these novel biological drugs [25,26]. Among their wide range of functions, both IMiDs are able to alter the stromal dependence of myeloma cells. Thalidomide, in particular, has been assumed to induce a change in the expression of adhesion molecules and the loss of leukocyte-associated antigens in myeloma cells. This leads to plasma cell dedifferentiation in plasmablasts (with the disappearance of the M component) and extramedullary progression [25]. 

The detection of new oligoclonal components showing an immunoglobulin isotype switch after autologous or allogenic SCT has also been described. However, this phenomenon does not identify a relapse and is not related to extramedullary progression; it represents a transient, benign event (also called secondary MGUS), which suggests robust immune reconstitution and predicts a better prognosis [27,28]. 

Most probably, our patient selected a further clone with different monoclonal secretion having previously reached CR after transplants and LENA maintenance. New clone selection is often associated with new mutations, cytogenetic alterations, and clinical manifestations that demonstrate the aggressiveness of the disease and lead to drug resistance. Very sadly, our patient passed away in these last few days.

## 4. Conclusions

EMD remains a therapeutically and biologically challenging issue. The pathogenetic mechanisms are not fully understood, and they are generally associated with high-risk cytogenetics, which in itself portends poor outcomes. In the era of new agents, an increasing incidence of EMD has been recorded, probably a reflection of a longer OS without a standard therapeutic approach. Patients benefit from aggressive chemotherapy-based approaches, but OS and prognosis remain poor. Unfortunately, to date, patients with EMD remain underestimated, and above all, they are a therapeutic challenge. There is therefore a need for large prospective studies to develop therapeutic approaches useful for the treatment of EMD. Lastly, better characterization of underlying biochemical patterns can allow a better risk stratification of this disease, leading to a more precise approach and proving the unvaluable cooperation between clinical and biological research. 

## Data Availability

Data are contained within the article.
